# Effect of SOX2 Repression on Corneal Endothelial Cells

**DOI:** 10.3390/ijms21124397

**Published:** 2020-06-20

**Authors:** Jin Sun Hwang, Ho Chul Yi, Young Joo Shin

**Affiliations:** 1 Department of Ophthalmology, Hallym University College of Medicine, 1, Hallymdaehak-gil, Chuncheon-si, Gangwon-do 24252, Korea; hotsayme@naver.com (J.S.H.); blissdoctor@gmail.com (H.C.Y.); 2 Department of Ophthalmology, Hallym University Medical Center, 1 Shingil-ro, Youngdeungpo-gu, Seoul 07441, Korea

**Keywords:** SOX2, human corneal endothelial cells, WNT signaling

## Abstract

Purpose: Human corneal endothelial cells (hCECs) pump out water from the stroma and maintain the clarity of the cornea. The sex-determining region Y-box 2 (SOX2) participates in differentiation during the development of the anterior segment of the eye and is found in the periphery of wounded corneas. This study was performed to investigate the effect of SOX2 repression on hCECs. Methods: Cultured hCECs were transfected by siRNA for *SOX2*. The wound healing rate and cell viability were measured. The cell proliferation-associated protein level was evaluated by Western blotting and RT-PCR. The energy production and mitochondrial function were measured, and cell shape and WNT signaling were assessed. Results: Upon transfecting the cultured cells with siRNA for *SOX2,* the SOX2 level was reduced by 80%. The wound healing rate and viability were also reduced. Additionally, CDK1, cyclin D1, SIRT1, and ATP5B levels were reduced, and CDKN2A and pAMPK levels were increased. Mitochondrial oxidative stress and mitochondrial viability decreased, and the cell shape became elongated. Furthermore, SMAD1, SNAI1, WNT3A, and β-catenin levels were increased. Conclusion: SOX2 repression disrupts the normal metabolism of hCECs through modulating WNT signaling and mitochondrial functions.

## 1. Introduction

Human corneal endothelial cells (hCECs) form the inner hexagonal shaped layer of the cornea [1]. Their apical surfaces are in contact with the aqueous humor in the anterior chamber and their basement membrane, termed Descemet’s membrane, is located on the inner stroma [2]. The lateral intercellular junctions are quite loose and leaky, such that aqueous humor diffuses into the stroma. hCECs pump out water from the stroma and maintain the clarity of the cornea [2]. hCECs have been reported to lack proliferative activity in vivo [3]. During the wound healing process, hCECs respond by the enlargement and migration of adjacent cells [4]. The hCECs react with compensatory cellular hypertrophy [5] during the wound healing process and fail if the functions of hCECs no longer compensate [4]. The decompensated cornea is swollen, blistered, and painful [6].

Endothelial-mesenchymal transition (EndoMT) has been suggested to promote fibrosis and is recognized as a novel mechanism for the generation of myofibroblasts [7]. During EndoMT, cells lose their shape and apical-basal polarity to become elongated and spindle-shaped mesenchymal cells and become non-adherent and migratory [7]. The shape of hCECs is important because the hexagonal shape is essential to maintain their functions [1]. Morphological changes including the loss of hexagonality is the marker for corneal endothelial disorder [8,9].

The sex-determining region Y-box 2 (SOX2) is a transcription factor that regulates self-renewal or pluripotency in stem cells [10]. SOX2 is essential for the initiation of EndoMT through the activation of specific serine proteases [11]. However, SOX2 participates in differentiation during the development of the anterior segment of the eye and is found in the periphery of wounded corneas [12]. SOX2 activation using CRISPR/dCAS9 system has been reported to regenerate the corneal endothelium [13]. However, the role of SOX2 repression in hCECs has not been reported. In this study, we investigated the effect of SOX2 repression on cultured hCECs.

## 2. Results

### 2.1. Cell Culture and Transfection

**Figure 1:**
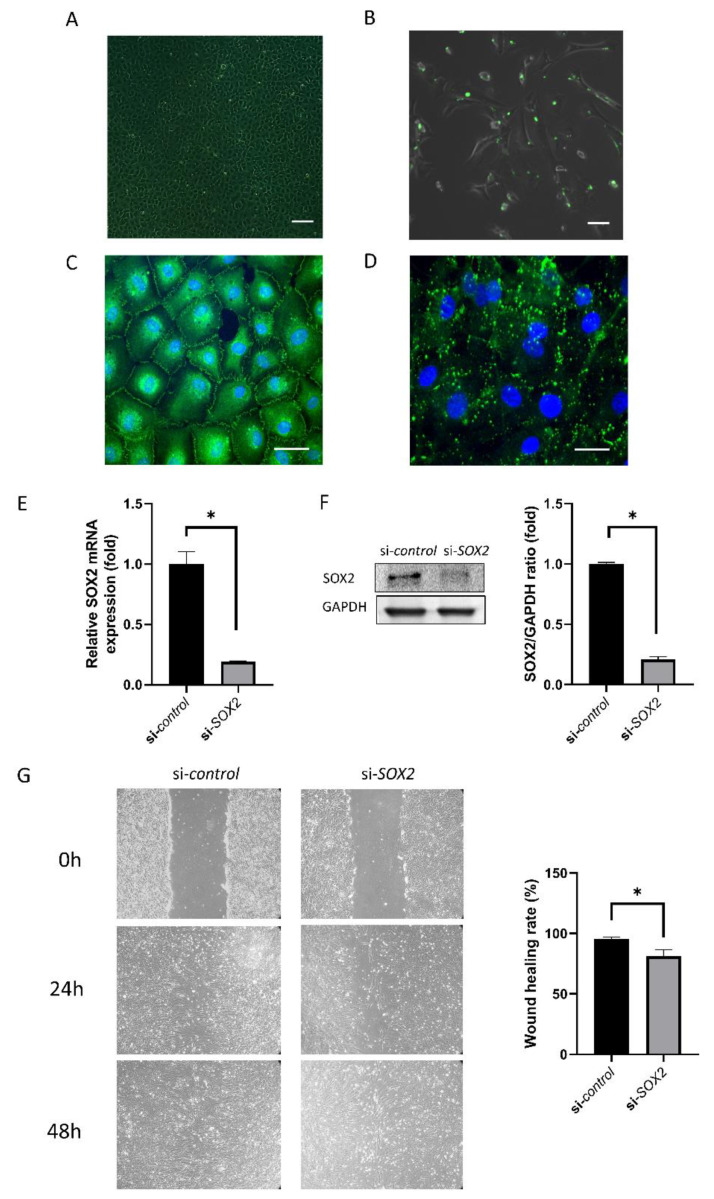
Human corneal endothelial cell (hCEC) culture and transfection of siRNA. (**A**) hCECs cultured at P0. The scale bar denotes 200 μm. (**B**) Green fluorescence of transfected cells indicates fluorescein isothiocyanate-conjugated control siRNA at 48 h after transfection. The confluency of the cells at the time of transfection should be about 70%. The scale bar denotes 100 μm. (**C**) Immunostaining for zonula occludens-1 (ZO-1). Green is ZO-1 and blue is Hoechst 33,342 nuclear staining. The scale bar denotes 50 μm. (**D**) Immunostaining for zonula connexin 43 (Cx43). Green is Cx43 and blue is Hoechst 33,342 nuclear staining. The scale bar denotes 50 μm. (**E**,**F**) sex-determining region Y-box 2 (SOX2) level was evaluated using RT-PCR and Western blotting. (**G**) Scratch assay monitoring the wound healing rate. Wound healing was delayed in si*-SOX2*-transfected cells. * indicates statistical significance by independent *t*-test.

hCECs were cultured (Figure 1A) and transfected with a transfection efficiency was 80.67 ± 2.31% (Figure 1B). Immunofluorescence staining for ZO-1 and Cx43 showed the distribution of ZO-1 and Cx43 (Figure 1C,D). SOX2 mRNA expression was reduced by 79.04 ± 0.02% in si-*SOX2*-transfected cells (Figure 1E), which was confirmed by Western blotting (Figure 1F). The wound healing rate was 95.30 ± 1.71% in si-control and 81.24 ± 5.39% in si-*SOX2*-transfected cells (*p* = 0.003; Figure 1G).

### 2.2. EndoMT and WNT Signaling

**Figure 2:**
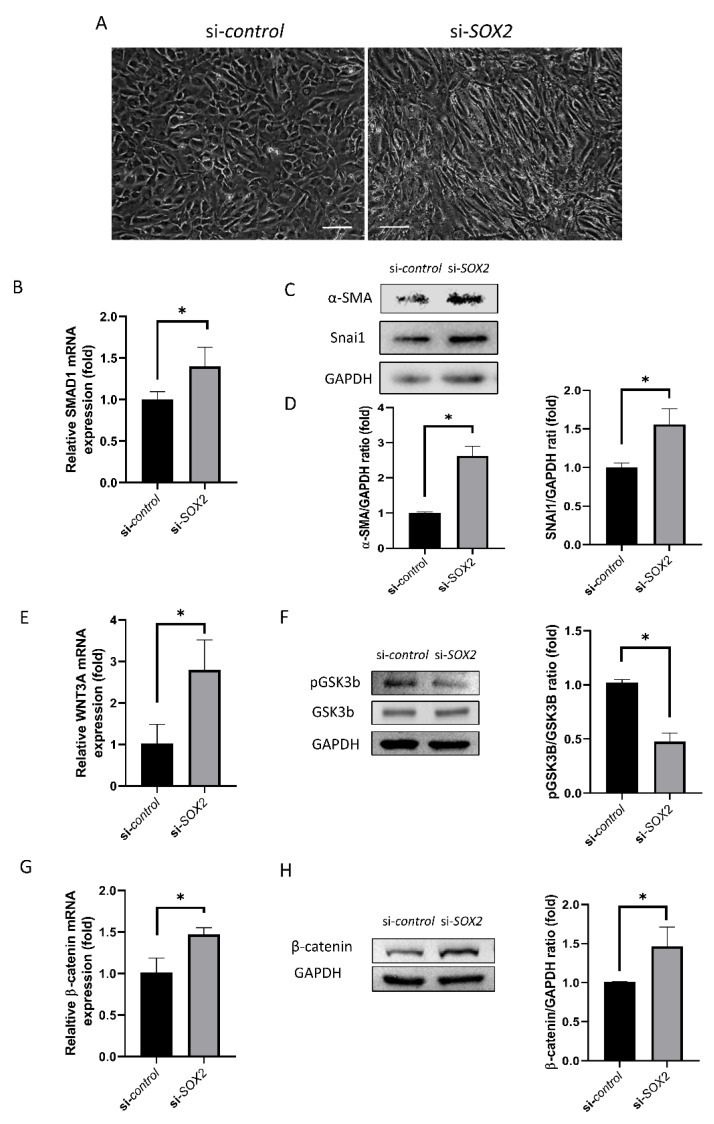
Endothelial-mesenchymal transition and WNT signaling pathway. (**A**) Cell shape in si-control and si-SOX2 cells. Scale bar denotes 150 μm. (**B**) SMAD1 mRNA expression. (**C**) α-SMA level determined by Western blotting. (**D**) SNAI1 level determined by Western blotting. (**E**) WNT3A mRNA expression. (**F**) GSK3β activation determined by Western blotting. (**G**,**H**) β-catenin level determined by RT-PCR and Western blotting. * indicates statistical significance by independent *t*-test.

*si-SOX2*-transfected cells showed slender, elongated and bipolar shape with thin cytoplasm (Figure 2A). Compared to si-control, relative SMAD1 mRNA expression was higher in si*-SOX2*-transfected cells (40%, *p* = 0.024; Figure 2B). α-SMA level and SNAI1 level was increased in si*-SOX2*-transfected cells (61.3% and 56%, *p* = 0.008 and 0.014; Figure 2C,D).

Relative WNT3A mRNA expression was higher in si-*SOX2*-transfected cells compared to si*-*control (*p* = 0.026; Figure 2E), while pGSK3B level was reduced (*p* < 0.001; Figure 2F). Additionally, relative β-catenin mRNA expression was higher in in si*-SOX2*-transfected cells (47%, *p* = 0.015; Figure 2G) which was confirmed by Western blotting (*p* = 0.033; Figure 2H).

### 2.3. Cell Viability and Proliferation

**Figure 3:**
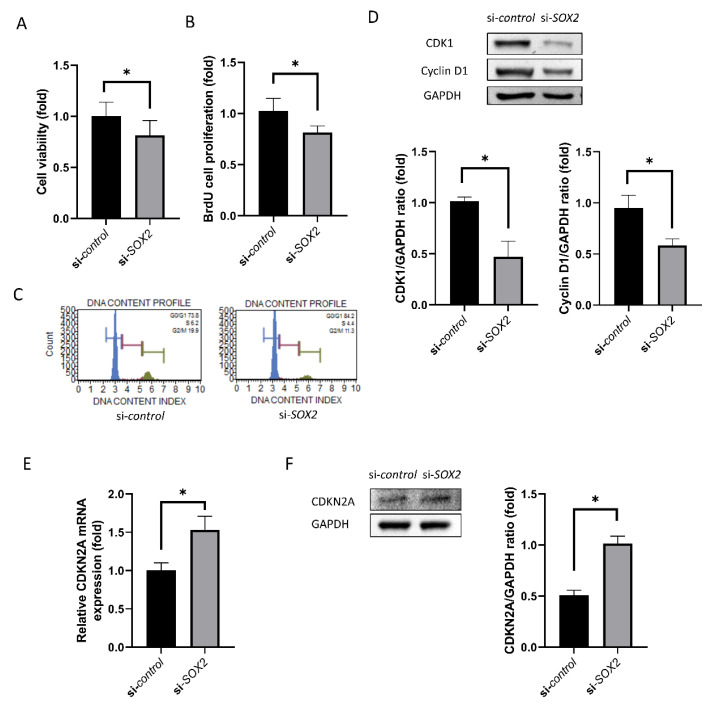
Cell viability and proliferation. (**A**) Cell viability. (**B**) BrdU cell proliferation rate. (**C**) Cell cycle analysis. (**D**) CDK1 and cyclin D1 levels. (**E**) CDKN2A mRNA expression evaluated by RT-PCR. (**F**) CDKN2A level evaluated by Western blotting was higher in si*-SOX2*-transfected cells. Graph provides the results from triple experiments. * indicates statistical significance by independent *t*-test.

Cell viability and BrdU cell proliferation rate was lower in si*-SOX2*-transfected cells compared to si-control (19% and 21%, *p* = 0.029 and 0.009; Figure 3A,B). Cell cycle analysis showed that the percentage of cells in S-phase was lower in si-*SOX2*-transfected cells compared to si-control (Figure 3C). In addition, CDK1 and cyclin D1 amounts were lower in si*-SOX2*-transfected cells (54% and 37%, *p* = 0.004 and 0.001; Figure 3D). Contrastingly, CDKN2A mRNA expression was higher in si-*SOX2*-transfected cells (53%, *p* = 0.004; Figure 3E), which was confirmed by Western blotting (Figure 3F).

### 2.4. Mitochondrial Functions

**Figure 4:**
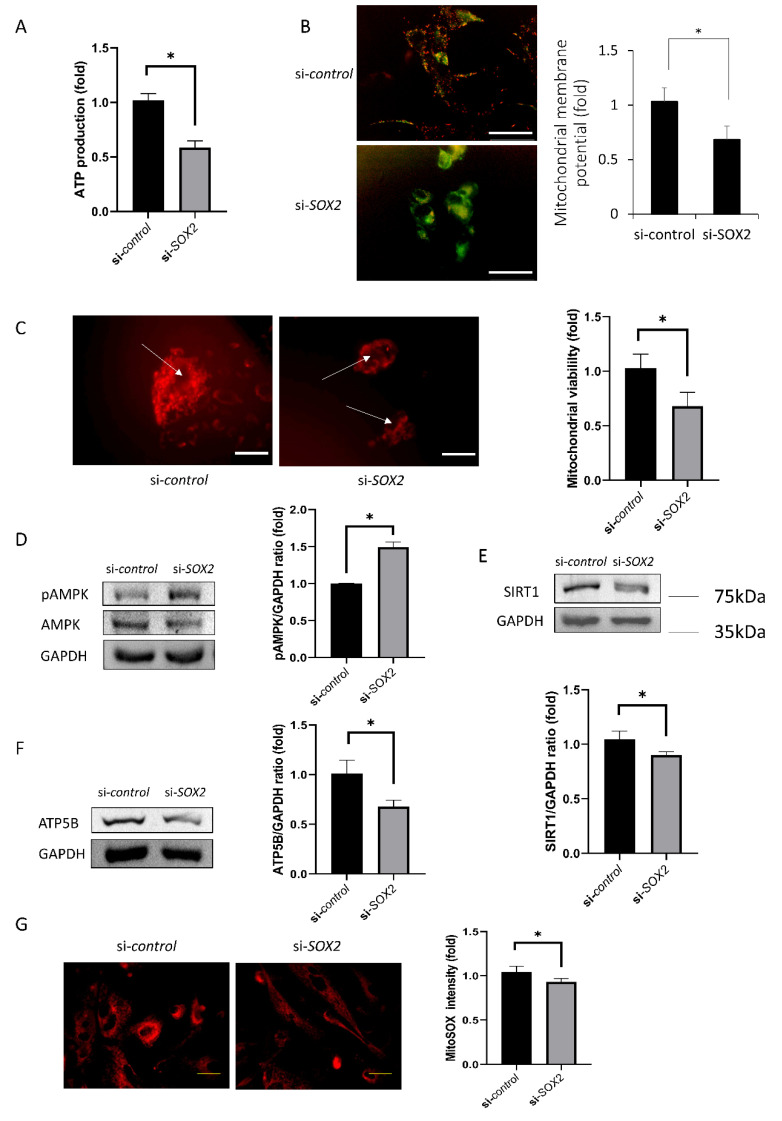
Mitochondrial functions. (**A**) Energy production in hCECs. (**B**) Mitochondrial membrane potential using the JC-1 probe. Scale bar denotes 100 μm. (**C**) Mitochondrial viability. Scale bar denotes 75 μm. Arrows indicating nuclei. (**D**) pAMPK level. (**E**) SIRT1 level. (**F**) ATP5B amount was lower in si*-SOX2*-transfected cells. Graph provides the results from triple experiments. (**G**) Mitochondrial oxidative stress levels by MitoSOX fluorescence intensity. Scale bar denotes 50 μm. * indicates statistical significance by independent *t*-test.

ATP production and mitochondrial membrane potential decreased in si-*SOX2*-transfected cells compared to si-control (85% and 32%, *p* = 0.006 and 0.003, Figure 4A,B). Mitochondrial viability decreased in si*-SOX2*-transfected cells compared to si*-*control (42%, *p* < 0.001, Figure 4C). pAMPK level increased in si-*SOX2*-transfected cells compared to si-control (50%, *p* < 0.001; Figure 4D), while SIRT1 and ATP5B levels decreased (14% and 32%, *p* = 0.034 and 0.018, respectively; Figure 4E,F). Mitochondrial oxidative stress levels also decreased (7%, *p* = 0.015; Figure 4G).

## 3. Discussion

hCECs play an essential role in maintaining the dehydration of the cornea, leading to corneal transparency [14]. However, details of the wound healing and regeneration of corneal endothelium have remained unclear because hCECs do not proliferate in vivo. SOX2 is expressed in cells at the wound margin during wound healing of corneal endothelium and during the development of corneal endothelium in the fetus [12]. SOX2 has been reported to induce proliferation and migration of somatic cells through reprogramming. [15] In this study, we investigated the role of SOX2 in hCECs through the repression of SOX2.

Cx43 and ZO-1 is used as a differentiation marker [16,17]. Cx43 is a main component in the gap junction [18]. Cx43 expression pattern alters dynamically and its knockdown promotes wound healing of corneal endothelium [19]. ZO-1 is a tight junction protein that is usually located at cell-cell adhesion membrane complexes [20].

We cultured hCECs and conducted siRNA transfection. FITC-conjugated siRNA was used to evaluate transfection efficiency. SOX2 level was reduced in si-*SOX2*, which was confirmed using RT-PCR and Western blotting. Wound healing was inhibited in the si*-SOX2* group compared to control. SOX2 has been linked to EndoMT, which was also evaluated. The cell shape changed and became similar to the shape of mesenchymal cells. SMAD1 level increased. SMAD1 signaling is required for the induction of EndoMT [21]. SMAD1 is regulated by Wnt signal activation through changing Wif1 expression [22]. SOX2 binds to SNAI1, and SNAI1 level is regulated by a canonical Wnt-GSK3β pathway [23]. The present data revealed that SOX2 repression induces the activation of the Wnt signaling pathway. WNT3A and β-catenin levels increased, and pGSK3β level was reduced. WNT3A, β-catenin, and pGSK3β are components of the Wnt signaling pathway [24], and the activation of this pathway reportedly induces EndoMT [24,25]. WNT3A is a Wnt protein that activates the canonical Wnt pathway and to be bioactive as determined by TCF/LEF [26]. WNT3A involved in neural crest cell differentiation and hCECs are derived from neural crest [27]. hCECs maintain the homeostasis in response to WNT3A [28]. It is not clear whether hCECs secrete WNT3A, but hCECs respond to WNT3A [28], and this study showed that the inhibition of SOX2 increased WNT3A. Wnt1 also controls neural crest cells migration and differentiation [29]. Further studies are needed for the response of hCECs to WNT1. SOX2 is a well-known regulator of neural progenitors [30]. SOX2 modulates WNT3A/β-catenin signaling pathway [28]. SOX2 inhibits Wnt-β-catenin signaling [31]. β-catenin plays a critical structural role in cadherin-based adhesions and is also an essential co-activator of Wnt-mediated gene expression [32]. The elevated expression of β-catenin may enforce the cell adhesion. β-catenin is present in the cell membrane, and when wnt signaling is activated, it enters the nucleus and activates wnt-related transcription factors [33]. WNT signaling cannot be suppressed in SOX2-deficient cells because SOX2 inhibits WNT signaling [13].

In this study, cell viability and cell proliferation-associated protein levels were reduced in the si*-SOX2* group. CDK1 and cyclin D1, which have been reported to increase during proliferation [34,35], were elevated. CDKN2A, which is an inhibitor of proliferation [36], was reduced. Cell proliferation and migration require energy production and the enhancement of mitochondrial function [37]. In this study, ATP production, mitochondrial viability, ATP5B level, and SIRT1 level were reduced and pAMPK level was increased. Mitochondrial activity and metabolic ATP production is essential for function, survival and proliferation. hCECs are metabolically active because they actively pump out the water from the stroma using ATP [38]. Mitochondrial membrane potential and oxidative stress levels were decreased. JC-1 and mitochondrial viability staining provide the visible fluorescence in only living cells and thus nuclear counter staining was not performed because DAPI or propidium iodide for nuclear staining do not permeate viable cell membranes [39]. ATP provides energy for cell functions and is produced in the mitochondria [37]. ATP5B is an ATP synthase present in mitochondria [40]. AMPK is activated when intracellular ATP levels drop and this allows the cells to adapt to low-energy conditions [41]. SIRT1, deacetylates histones, are involved in chromatin modification and gene silencing, and are also associated with aging and longevity [42]. Mitochondrial membrane potential is required for ATP production [43]. The loss of mitochondrial membrane potential results in ATP depletion and modulates mitochondrial oxidative stress levels [40,44]. The limitation of this study is that an animal study was not performed. Although in vitro studies are faster and easier to perform and quantify, in vitro studies can be criticized for being very different from the natural environment [45]. Further study to investigate the role of si*-SOX2* using an in vivo model would be necessary.

In conclusion, SOX2 repression disrupts the normal functions of hCECs by modulating the Wnt/mitochondrial pathway.

## 4. Materials and Methods

### 4.1. Cell Culture and Transfection

This study was performed according to the tenets of the Declaration of Helsinki and was reviewed and approved by the institutional review board/ethics committee of Hallym University Medical Center (2018-07-020, 3 August 2018). The corneas were purchased from Lion eyebank (Portland, OR, USA), with informed consent for all tissues. The cells were cultured based on previously published methods [46,47]. The corneas from a total of six donors were used [46]. hCECs obtained from the remnant donor limbal tissue after corneal transplantation were harvested on or before the seventh day after death. All of the cells remained attached to the Descemet’s membrane. Descemet stripping was performed carefully under a surgical microscope with the CECs side up, and only CEC-Descemet’s membrane complex was obtained. The CECs remained attached to Descemet’s membrane and incubated in media overnight at 37 °C. The morphology and shape of CECs were confirmed during harvest and at p0. The CEC—Descemet’s membrane complex—was incubated for 10 min in 0.25% trypsin/0.02% ethylenediaminetetraacetic acid (EDTA) solution. After centrifuging at 1500 rpm at 25 °C for 3 min, the supernatant was removed and the cell pellet was resuspended in the media. The cells were then plated at the bottom of the wells of 6-well plates coated with a fibronectin-collagen combination (FNC) coating mix (Athena Environmental Sciences, Inc., Baltimore, MD, USA). The cells were then cultured in OptiMem-I media (GIBCO/BRL Life technologies, Grand Island, NY, USA) supplemented with 8% FBS (Cambrex Bio Science, Walkersville, MD, USA), 200 mg/L of calcium chloride (Sigma Chemical Co., St. Louis, MO, USA), 0.08% chondroitin sulfate (Sigma Chemical Co.), 20 μg/mL ascorbic acid (Sigma Chemical Co.), 100 μg/mL pituitary extract (Invitrogen, Grand Island, NY, USA), 5 ng/mL epidermal growth factor (Sigma Chemical Co.), 20 ng/mL nerve growth factor (Sigma Chemical Co.), 10 μg/mL gentamicin (Invitrogen), 100 IU/mL penicillin (Cambrex Bio Science), 100 IU/mL streptomycin (Cambrex Bio Science), and 2.5 μg/mL amphotericin (Cambrex Bio Science) under an atmosphere of 5% CO_2_. The medium was changed every 2 days. Cells were cultured for 14–21 days until confluency and were then passaged at a ratio of 1:3 using 0.25% trypsin/0.02% EDTA solution.

To evaluate the transfection efficiency, fluorescein isothiocyanate (FITC)-conjugated siRNA were transfected to the cells. To silence *SOX2*, we used small-interference RNA (siRNA). siRNA for *SOX2* (sense, 5′-GCA GCU GAA AUU UAG GAC A-3′ and antisense, 5′-UGU CCU AAA UUU CAG CUG C-3′) and non-specific control siRNA (SN-1001) used as a negative control, were purchased from Bioneer Cooperation (Daejeon, CA, USA). Summarily, primary hCECs at a density of 5 × 10^4^ cells/cm^2^ were transfected with siRNA specific for *SOX2* at 10 nM concentrations, with a non-coding sequence siRNA as a negative control, using Lipofectamine™ RNAiMAX (Invitrogen, Carlsbad, CA, USA) according to the manufacturer’s instructions. The transfections were performed at 70% confluency. After incubation for 48–72 h, the cells were collected for further evaluation. The cells were separated into two groups: siRNA group targeting *SOX2* (si-*SOX2*), and a control group (si-control). The effect of *SOX2* silencing was confirmed by Western blot analysis or RT-PCR 48 h after transfection.

### 4.2. Immunofluorescence Staining for Zonula Occludens-1 (ZO-1) and Connexin 43 (Cx43)

Cultured non-transfected hCECs were immunostained for ZO-1 and Cx43. Briefly, the cells were cultured on cover glasses in 12-well plates, washed with PBS, and fixed for 20 min in using 4% paraformaldehyde in PBS. The cells were permeabilized in 0.3% triton X-100 for 10 min, blocked with 5% skim milk at room temperature for 30 min, incubated overnight with rabbit anti-ZO-1 (sc-10804, Santa Cruz Biotechnology, Santa Cruz, CA, USA) or rabbit anti-Cx43 antibody (ab11370, Abcam, Cambridge, UK) at 4 °C, and then treated with fluorescein isothiocyanate-conjugated goat anti-rabbit antibody for 2 h. After nuclear counterstaining with Hoechst nuclear staining dye (1:2000; Molecular Probes, Eugene, OR, USA), the slides were mounted in a drop of mounting medium to reduce photobleaching. The slide was observed by fluorescence microscope (Leica DMi8, Leica Microsystems, Wetzlar, Germany).

### 4.3. Cell Viability and Proliferation

Cells (1 × 10^4^) were cultured in a 96-well plate. Cell viability was measured using a cell counting kit-8 (CCK-8; Dojindo, Kumamoto, Japan) based on the water-soluble monosodium tetrazolium salt, WST-8 [2-(2-methoxy-4-nitrophenyl)-3-(4-nitrophenyl)-5-(2,4-disulfophenyl)-2H-tetrazolium]. The plates were incubated with CCK-8 solution for 1–2 h. Cell viability was determined by measuring the absorbance at 450 nm using a microplate spectrophotometer. Cell viability was expressed as the mean ± standard deviation, as a percentage of the control (100%). Experiments were repeated three times, and a representative experiment is shown.

Cell proliferation rate was measured using a commercial bromodeoxyuridine (BrdU) proliferation assay kit (Roche Diagnostics, GmbH, Mannheim, Germany) according to the manufacturer’s protocol. Briefly, cells (5 × 10^3^ cells/well) were placed in 96-well plates and incubated for 48 h in a humidified atmosphere containing 5% CO_2_. After incubating the plate in the FixDenat solution for 30 min at 25 °C, the cells were incubated with anti-BrdU-peroxidase solution for approximately 90 min at room temperature. Next, the substrate solution was added to each well, and the plate was incubated for 20 min at room temperature. Subsequently, 1 M H_2_SO_4_ was added to each well to stop the reaction. The optical density was measured at 450 nm using an ELISA reader. Proliferation rates were expressed as the percentage of controls after subtraction of the corresponding blanks.

### 4.4. Wound Healing Assay

Cells were seeded in 96 well plates. After 48 h, the cell monolayer was scrapped with 96-pin IncuCyte WoundMaker Tool (Essen BioScience, Ann Arbor, MI, USA). The cells were washed once with the medium to remove the debris and then treated with siRNA. Images were obtained at 24 h and 48 h. Wound healing rate were calculated by percentage of the cell covered area/total wounded area. Wounding areas were measured using AxioVision software (Carl Zeiss AG, Oberkochen, Germany) after drawing the wounding boundaries. Three regions were evaluated. The size of initial scratch was the same between control and si-SOX2. The scratch assay has been commonly used as wound healing assay. The scratch assay is cost-effective and requires minimal equipment although reducing accuracy [48].

### 4.5. ATP Production and Mitochondrial Oxidative Stress Measurement

The ADP/ATP Ratio Bioluminescence Assay Kit (Biovision, San Francisco, CA, USA) was used to evaluate ATP production in mitochondria Cells (2.5 × 10^5^) were lysed in a buffer (containing 20 mM Tris, pH 7.0; 0.5% NP-40; 25 mM NaCl; 2.5 mM EDTA; and 2.5 mM EGTA). Immediately, the samples were placed in a white 96-well plate and the reaction solution was added. After 5 min, luminescence was measured in an LMax™ microplate luminometer (Molecular Devices Corp., Sunnyvale, CA, USA). The relative ATP levels were normalized with the protein concentration measured using the BCA Protein Assay kit (Thermo Fisher Scientific, Waltham, MA, USA). Mitochondrial membrane potential was measured using the JC-1 probe (Invitrogen, Carlsbad, CA, USA). Cells seeded onto black 96-well plates were treated with siRNA at 37 °C for 48 h. JC-1 dye was used to determine the changes in the ΔΨ_m_ of hCECs. The cells were incubated with a final concentration of 1 μM JC-1 for 30 min at 37 °C in the dark. Each well was analyzed by a spectrofluorometer (SFM 25, Kontron Instruments, Everett, MA, USA). Measurement of fluorescence intensity in each well was conducted at an excitation/emission wavelength of 495 nm/530 nm for the JC-1 monomer, and an excitation/emission wavelength of 525 nm/590 nm for JC-1 aggregates. ΔΨ_m_ was calculated by a red/green fluorescence ratio of JC-1. The relative intensity of fluorescence was calculated by setting the fluorescence intensity of the non-treated cultures to 100% after subtracting the corresponding blanks. MitoSOX Red (final concentration, 2.5 μM; Invitrogen, Carlsbad, CA, USA) was used to measure mitochondrial oxidative stress. The cells were incubated with 5 μM MitoSOX^TM^ reagent for 10 min at 37 °C in the dark. Fluorescence intensity in each well was measured at an excitation wavelength of 525 nm and an emission wavelength of 590 nm. The relative intensity of MitoSOX^TM^ Red fluorescence was calculated.

Mitochondria viability was measured using mitochondrial viability stain (ab129732, Abcam). It measures oxidation-reduction reactions which principally occur in the mitochondria of live cells. Cells were seeded in a black 96-well plate and incubated at 37 °C for 4 h after overlaying 100 µL of 2× mitochondrial viability stain solution on each well, containing 100 µL of media, and then washed with PBS. Fluorescence intensity was measured at an excitation wavelength of 525 nm and emission wavelength of 595 nm. The relative intensity of fluorescence was calculated and normalized by cell numbers.

### 4.6. Western Blotting

Radioimmunoprecipitation assay buffer (Biosesang, Seoul, Korea) containing a protease inhibitor cocktail (Sigma-Aldrich, St. Louis, MO, USA) and phosphatase inhibitor cocktail (PhosSTOP; Roche, Basel, Switzerland) was used for isolation of total cellular proteins. Western blotting was conducted using standard protocols. A 5% solution of skim milk or gelatin was used to block the nonspecific binding for 1 h. Primary antibodies used were rabbit anti-human GAPDH antibody (LF-PA0212, Abfrontier, Seoul, Korea; 1:5000 dilution), rabbit anti-human SOX2 antibody (sc-365823, Santa Cruz Biotechnology; 1:500 dilution), rabbit anti-human CDK1 antibody (ab131450, Abcam; 1:1000 dilution), rabbit anti-human cyclin D1 antibody (sc-718, Santa Cruz Biotechnology; 1:1000 dilution), anti-cyclin-dependent kinase Inhibitor 2A antibody (CDKN2A; MABE1328, Merck Millipore, Burlington, MA, USA, 1:500 dilution), rabbit anti-human AMP-activated protein kinase antibody (AMPK; sc-25792, Santa Cruz Biotechnology; 1:1000 dilution), rabbit anti-human phospho-AMPK antibody (pAMPK; sc-101630, Santa Cruz Biotechnology; 1:1000 dilution), rabbit anti-human SIRT1 antibody (sc-15404, Santa Cruz Biotechnology; 1:1000 dilution), mouse anti-ATP synthase subunit β antibody (ATP5B; sc-55597, Santa Cruz Biotechnology; 1:1000 dilution), mouse anti-human α-SMA antibody (sc-133098, Santa Cruz Biotechnology; 1:1000 dilution), mouse anti-human SNAI1 antibody (sc-271977, Santa Cruz Biotechnology; 1:1000 dilution), rabbit anti-human pGSK3B antibody (sc-130601, Santa Cruz Biotechnology; 1:1000 dilution), rabbit anti-human GSK3B antibody (ab32391, Abcam; 1:1000 dilution), or rabbit anti-human β-catenin antibody (ab325572, Abcam; 1:1000 dilution). Horseradish peroxidase (HRP) conjugated secondary antibody and a Miracle-Star™ Western Blot Detection System (iNtRON Biotechnology, Seoul, Korea) were used for detecting immunoreactive bands. Data were quantified by video image analysis. Protein bands were measured by densitometry.

### 4.7. Real-Time Reverse Transcription PCR

Total RNA was extracted using the ReliaPrep RNA Miniprep System (Promega, Madison, WI, USA). cDNA was generated using the Promega GoScript reverse transcription system according to the manufacturer’s protocol. Real-time quantitative PCR was performed on a LightCycler 96 (Roche Diagnostics) using AccuPower^®^ 2× GreenStar™ qPCR Master Mix (SYBR Green; Bioneer, Seoul, Korea). All reactions were performed in triplicate, and data were analyzed according to the ΔΔCt method. The primer sequences used were as follows: β-actin (forward: 5′-AGAGCTACGCTGCCTGAC-3′; reverse: 5′-AGCACTGTTGGCGTACAG-3′), SOX2 (forward: 5′-GCCCTGCAGTACAACTCCAT-3′; reverse: 5′-TGGAGTGGGAGGAAGAGGTA-3′), CDKN2A (forward: 5′-CATAGATGCCG CGGAAGGT-3′; reverse: 5′-CTAAG TTTCCCGAGGTTTCTCAGA-3′), SMAD1 (forward: 5′-TACGCCCCCACCTGCTTAC-3′; reverse: 5′-TTGTGTCCATCGGCTGAGA-3′), WNT3A (forward: 5′-TCAGCTGCCAGGAGTGCACG-3′; reverse: 5′- CGCCCTCAGGGAGCAGCCTAC-3′), and β-catenin (forward: 5′-AAAGCGGCTGTTAGTCACTGG-3′; reverse: 5′-CGAGTCATTGCATACTGTCCAT-3′).

### 4.8. Cell Cycle Analysis

Cell cycle analysis was performed using the Muse cell analyzer (Merck Millipore, Burlington, MA, USA) with propidium iodide (PI) staining according to the manufacturer’s protocol. Briefly, cells were grown in 6-well plates and transfected with siRNA; then harvested by trypsinization and washed twice with PBS. The cells were fixed with 1 mL of 70% cold ethanol at −20 °C for 5 h and washed twice with PBS. After centrifugation at 1500 rpm for 5 min, the samples were treated with 200 uL solution including 50 μg/mL PI (Merck Millipore) and 100 μg/mL RNase A (Biosesang, Seongnam, Korea).

### 4.9. Statistics

Data were expressed as mean ± standard deviation. An independent *t*-test was used for comparison of two groups.
